# *Lactobacillus* as a rare cause of an infected total knee replacement: a case report

**DOI:** 10.4076/1752-1947-3-7441

**Published:** 2009-07-31

**Authors:** Navraj Atwal, Akintunde George, Ben Squires, Clayton H Marsh

**Affiliations:** 1 Department of Trauma and Orthopaedics, Bristol Royal Infirmary, Marlborough Street, Avon, BS2 8HW, UK; 2 Department of Trauma and Orthopaedics, Royal Devon Exeter NHS Trust, Barrack Road, Exeter, EX2 5DW, UK; 3 Department of Trauma and Orthopaedics, Musgrove Park Hospital, Taunton and Somerset NHS Trust, Taunton, TA1 5DA, UK

## Abstract

**Introduction:**

We report a rare case of an infected revision total knee replacement as a result of a *Lactobacillus* species infection. *Lactobacillus* infections have been associated with prolonged broad-spectrum antibiotic use. This can have implications in revision surgery, especially when patients have been on previous long-term suppressive antibiotic therapy.

**Case presentation:**

An 81-year-old British man with a previous history of complex revision knee arthroplasty for infection presented with a hot, swollen knee joint. He had previously been on long-term suppressive antibiotic therapy. Aspiration of the knee joint yielded a culture of *Lactobacillus* species.

**Conclusion:**

In patients undergoing revision joint arthroplasty, especially for previous infection, the presence of common and uncommon bacterial species must be excluded and eradicated before further surgical intervention.

## Introduction

*Lactobacillus* is a Gram-positive facultative anaerobic bacterium normally found in the mucosal surfaces of the mouth, the gastrointestinal tract and the genitourinary tract. Previous studies have shown it to be associated with endocarditis and bacteraemia [1]. This is the first report of a *Lactobacillus* infection in a prosthetic knee joint.

## Case presentation

Our patient, an 81-year-old man who had hypertension with peripheral vascular disease, initially underwent a total knee replacement (TKR) for osteoarthritis in 1991. He later had two further revision procedures in the following three years, first for presumed infection (although all culture results were negative) and then for component failure. In 1999, he underwent complex revision surgery in an adjacent hospital, requiring a massive distal allograft of the distal femur and impaction grafting of the tibial component. This was performed via a tibial tubercle osteotomy and a constrained condylar total knee replacement was implanted. Unfortunately, the allograft did not fuse to the host bone, although the joint was mobile.

In late 1996, our patient was admitted as an emergency case with an infected knee replacement. He was febrile (39.5°C) and blood tests revealed a raised C reactive protein of 184 mg/L and peripheral white cell count of 14×10^9^/L. Blood cultures were negative. Joint fluid and multiple tissue specimens taken intra-operatively from the joint and capsule were sent for microscopy, culture and sensitivity. Gram staining identified Gram-positive bacilli in all specimens, which on culture was later confirmed as *Lactobacillus paracasei* through phenotypic characterisation using the API CH50 biochemical identification kit (BioMérieux). He was treated with intravenous amoxicillin and then oral clindamycin. Initially, he responded well and his inflammatory markers improved. However, a couple of weeks later, his symptoms recurred and the decision was taken to proceed with an above knee amputation. There were no postoperative problems.

## Discussion

To our knowledge, this is the first report of a *Lactobacillus*-associated infected total knee replacement. A recent case report identified lactobacillus as a cause of septic arthritis in a native joint [2] which required surgical intervention and antibiotic treatment. Only one case of prosthetic joint infection exists in the literature; it was unspecified which joint was affected and was reported as a *Lactobacillus* species infection [3]. No details of therapy or outcome were given.

*Lactobacillus* has been increasingly reported as a cause of serious infections in both immunocompetent and immunocompromised patients [4]. The species *L. casei* and *L. rhamnosus* are the most commonly identified causal organisms [1]. Lactobacilli are found primarily in the gastrointestinal tract and oral cavity. Food reservoirs include dairy products and probiotic preparations. *Lactobacillus* infections are predominantly seen in immunocompromised patients including those with diabetes, those on immunosuppressive therapy and those with underlying malignancy. Other risk factors include persistent neutropenia, use of broad-spectrum antibiotics, especially vancomycin, and gastrointestinal operations which may alter bowel flora [4].

In our case, the patient had no underlying medical conditions predisposing him to possible infections. There was no history of excessive probiotic ingestion. However, there was a history of previous vancomycin therapy associated with his multiple revision operations.

The majority of infections published involve Lactobacillaemia, with only two reports of isolated joint infections. Chanet et al. presented a patient with lactobacillus-associated septic arthritis in a native joint which was resistant to intravenous antibiotic therapy and eventually required surgical intervention [2]. Intravenous and oral antibiotic therapy has been shown to be effective for Lactobacillaemia without joint involvement [5]. Based on this, it would appear that antibiotic therapy alone is not indicated for joint sepsis.

## Conclusion

Patients undergoing revision joint surgery, especially those who have been on previous antibiotic therapy, are at risk of colonisation with atypical microorganisms. Therefore, one must consider common and uncommon bacterial infections as a potential cause of septic arthritis. Their eradication before definitive surgery is essential.

## Consent

Written informed consent was obtained from the patient for the publication of this case report. A copy of the written consent is available for review by the Editor-in Chief of this journal.

## Competing interests

The authors declare that they have no competing interests.

## Authors' contributions

NA was involved in the conception of the case report, wrote up the initial draft and reviewed the final manuscript. AG carried out the literature review, took part in the manuscript review and submission of the final script. BS and CHM were involved in assisting the conception of the case report, helped in the review and approval of the final manuscript.
